# Correlated physical and mental health summary scores for the SF-36 and SF-12 Health Survey, V.1

**DOI:** 10.1186/1477-7525-5-54

**Published:** 2007-09-07

**Authors:** Sepideh S Farivar, William E Cunningham, Ron D Hays

**Affiliations:** 1 Global Health Economics, Amgen, Inc., Thousand Oaks, CA, USA; 2 UCLA, School of Public Health, Los Angeles, CA, USA; 3 UCLA, Department of Medicine, Los Angeles, CA, USA; 4 RAND, Health Program, Santa Monica, CA, USA

## Abstract

**Background:**

The SF-36 and SF-12 summary scores were derived using an uncorrelated (orthogonal) factor solution. We estimate SF-36 and SF-12 summary scores using a correlated (oblique) physical and mental health factor model.

**Methods:**

We administered the SF-36 to 7,093 patients who received medical care from an independent association of 48 physician groups in the western United States. Correlated physical health (PCS_c_) and mental health (MCS_c_) scores were constructed by multiplying each SF-36 scale z-score by its respective scoring coefficient from the obliquely rotated two factor solution. PCS_c_-12 and MCS_c_-12 scores were estimated using an approach similar to the one used to derive the original SF-12 summary scores.

**Results:**

The estimated correlation between SF-36 PCS_c _and MCS_c _scores was 0.62. There were far fewer negative factor scoring coefficients for the oblique factor solution compared to the factor scoring coefficients produced by the standard orthogonal factor solution. Similar results were found for PCS_c_-12, and MCS_c_-12 summary scores.

**Conclusion:**

Correlated physical and mental health summary scores for the SF-36 and SF-12 derived from an obliquely rotated factor solution should be used along with the uncorrelated summary scores. The new scoring algorithm can reduce inconsistent results between the SF-36 scale scores and physical and mental health summary scores reported in some prior studies.

(Subscripts C = correlated and UC = uncorrelated)

## Background

Health-related quality of life (HRQOL) refers to functioning and well-being in physical, mental and social dimensions of life. The SF-36 and the SF-12 are the most frequently used multi-item HRQOL instruments [1,2]. The SF-36 is composed of 8 multi-item scales (35 items) assessing physical function (10 items), role limitations due to physical health problems (4 items), bodily pain (2 items), general health (5 items), vitality (4 items), social functioning (2 items), role limitations due to emotional problems (3 items) and emotional well-being (5 items) [1]. These eight scales can be aggregated into two summary measures: the Physical (PCS) and Mental (MCS) Component Summary scores [3]. The 36^th ^item, which asks about health change, is not included in the scale or summary scores. The SF-12 is a 12-item subset of the SF-36 that has two summary measures: the Physical (PCS-12) and Mental (MCS-12) Component Summary scores [2]. Higher scores represent better health.

**Figure 1:**
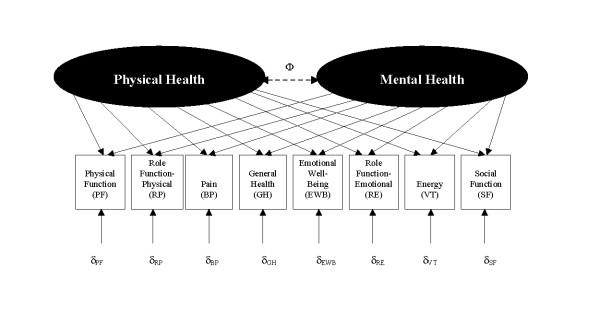
**Conceptual model for the SF-36 health survey**. Orthogonal (uncorrelated) model assumes the correlation between physical and mental health constructs is fixed at 0 (Φ = 0). Oblique (correlated) model allows correlation between the physical and mental health constructs. δ denotes error terms (uniqueness terms) associated with each scale. Directional associations exist between the physical and mental health and the 8 scales (as indicated by the arrows); however, the associations vary from large (e.g. physical functioning on physical health) to close to zero (e.g., emotional well-being on physical health).

The standard scoring algorithm for the SF-36 and SF-12 version 1 summary measures is based on a factor analytic technique that forces the scores to be orthogonal [2,3]. Figure 1 depicts the conceptual framework on which the orthogonal component summary scores are based. The model assumes that physical and mental health constructs are uncorrelated (Φ = 0). Recent studies have shown inconsistent results between the 8 SF-36 scale scores and the PCS and MCS [4-7]. For example, a study of 482 patients initiating antidepressant treatment found improvements from baseline to 3 months of 0.28–0.49 SD units on the physical health scales (physical functioning, role limitations due to physical health problems, pain, general health), but the PCSuc was essentially unchanged (from 51 to 50). These patients had large improvements on the emotional well-being scale (1.67 SD) [8].

Taft et.al. concluded that the discrepancies between results for the SF-36 scale scores and component scores are a result of the negatively weighted scales used in the PCS and MCS scoring algorithm [5,6]. The scoring algorithm for PCS includes positive weights for the physical functioning, role-physical, bodily pain, general health and vitality scales and negative weights for the social functioning, role-emotional and emotional well-being scales [3]. The scoring algorithm for MCS includes positive weights for the vitality, social functioning, role-emotional, and emotional well-being scales and negative weights for the physical functioning, role-physical, bodily pain and general health scales [3]. As such, higher mental health scale scores drive the PCS down and higher physical functioning scores drive the MCS down (and vice versa).

The objective of this study is to estimate the SF-36 summary scores (PCS_c _and MCS_c_) from a correlated (oblique) physical and mental health factor solution. In addition, we derive weights that can be used to create SF-12 component summary scores from the correlated factor model (PCS_c_-12 and MCS_c_-12). We hypothesize that the correlated factor model will produce better correspondence between the scale and summary scores. The results are compared to those obtained from the standard uncorrelated approach [3]. (Summary scores with a subscript "c" are based on oblique [correlated] factor analysis whereas summary scores with the subscript "uc" are created via orthogonal [uncorrelated] factor analysis.)

## Methods

### Sample

The sample consists of a random selection of patients receiving medical care from the Unified Medical Group Association (UMGA), an independent association of physicians in the western United States [9,10]. Patients were at least 18 years of age or older and had a minimum of one provider visit during the year prior to the data collection period from October 1994 to June 1995. Study participants were mailed $2 cash along with a 12-page questionnaire assessing HRQOL, patient evaluations of health care, utilization and demographic characteristics. Those who had not yet responded were sent a questionnaire two weeks later and were given a reminder telephone call. There were 7,093 respondents, a 59% response rate after adjusting for undeliverable surveys, ineligible respondents, and deceased. Our analysis was conducted on patients who had complete data for the SF-36 (n = 6,931).

### Deriving Weights for Correlated SF-36 PCS_c _and MCS_c_

The method used here is identical to that used by Ware et al. [3] except the factors were allowed to be correlated. Factor analysis of the 8 SF-36 scale scores with a two-factor oblique rotation was used to estimate the physical and mental health factor scoring coefficients (weights). PCS_c _was then constructed by multiplying each SF-36 scale z-score by its respective physical factor scoring coefficient and summing the eight products. Similarly, MCS_c _was created by multiplying each SF-36 scale z-score by its respective mental factor score coefficient and summing the products. The component scores were then transformed so that each had a mean of 50 and a standard deviation of 10 (T-score) in the sample.

### Sensitivity Analysis

In order to illustrate the potential differences in scores produced by the weights derived from the uncorrelated versus correlated factor analysis, we determined summary scores if the scales that load heavily on physical health (physical health, role physical, bodily pain, general health) have z-scores of 1 and the scales that load heavily on mental health (vitality, social functioning, role-emotional and emotional well-being) have z-scores equal to 0.3. Then we determined the summary scores if the z-scores for scales loading heavily on physical health are equal to 0.3 and z-scores for scales loading heavily on mental health are equal to 1.

### Deriving Correlated SF-12 PCS_c _and MCS_c_

To derive weights for the SF-12 summary measures, the SF-36 PCS_c _and MCS_c _were regressed in separate models on the SF-12 items. Dummy variables were created for each of the response choices of the 12 items, allowing the relationship of each level of each SF-12 item to vary rather than assuming a linear relationship. Following Ware et al. [2], the most favorable response choice for a question was the holdout category. As such, the parameters (weights) estimated are decrements associated with different SF-12 response choices. The predicted values in the models were the PCS_c_-12 and MCS_c_-12 scores, respectively.

## Results

Thirty-five percent of the sample was male. The majority was Caucasian (80%). The average age was 50 (SD = 18) The majority of the sample had either gone to vocational school, had some college, or completed college (55%) and had a household income greater than $20,000 (77%). Other sample characteristics and average scale and summary scores are given in Table 1. There were no differences between the demographic characteristics (gender, race, age, education, income) of the total respondent sample (n = 7,093) versus the analytic sample (n = 6,931). We also compared adult members in the sampling frame who visited the physician within the last 365 days (n = 1,203,001) and those who returned the questionnaire (n = 7,093). Those who returned a questionnaire tended to be slightly more likely to be older, female, to have hypertension, and to have visited the physician group more recently [10].

**Table 1:** Characteristics of respondent sample and analytic sample

	**Respondent Sample** **Frequency (Percent)**	**Analytic Sample** **Frequency (Percent)**
**Gender**		
Male	2,427 (35%)	2,389 (35%)
Female	4,487 (65%)	4,400 (65%)
**Race**		
Caucasian	5,508 (80%)	5,426 (80%)
Hispanic	713 (10%)	691 (10%)
Asian	298 (4%)	292 (4%)
African-American	210 (3%)	206 (3%)
Other	182 (3%)	172 (3%)
**Education**		
High school or less	2276 (33%)	2205 (33%)
College/Vocational	3731 (55%)	3685 (55%)
Professional/Graduate	834 (12%)	827 (12%)
**Income**		
≤ 9,999	525 (8%)	508 (8%)
10 – 19,999	966 (15%)	940 (15%)
20 – 39,999	1,949 (31%)	1,930 (31%)
40 – 74,999	2,070 (32%)	2,053 (32%)
75,000 +	889 (14%)	877 (14%)
**Insurance**		
Private	5,873 (86%)	5,787 (86%)
Medicaid	57 (1%)	54 (1%)
Medicare	398 (6%)	371 (6%)
Other Insurance	418 (6%)	409 (6%)
No Insurance	90 (1%)	89 (1%)
**Age**	50 (18)	50 (18)
**SF-36 Scale Scores**		
Physical Functioning	79 (27)	79 (27)
Role-Physical	72 (39)	73 (39)
Bodily Pain	69 (25)	70 (25)
General Health	70 (21)	70 (21)
Vitality	59 (21)	59 (21)
Social Functioning	79 (25)	79 (25)
Role-Emotional	81 (34)	81 (34)
Emotional Well-Being	75 (17)	75 (18)
**SF-36 Component Scores**		
PCS_uc_	47 (11)	47 (11)
MCS_uc_	50 (10)	50 (10)

* Sample size (n) vary because of missing data.

### Factor Analysis Results

The oblique two-factor solution indicated that role-physical (0.76), physical functioning (0.71), bodily pain (0.66) and general health (0.53) loaded heavily on the physical factor whereas emotional well-being (0.84), role-emotional (0.59), vitality (0.58) and social functioning (0.39) loaded most heavily on the mental factor. The estimated correlation between the two factors was 0.62 (Table 2).

**Table 2:** Primary factor pattern loadings for the two factor rotated (promax) solution and estimated correlation between factors

	**Physical**	**Mental**
Physical Functioning	0.71	-0.08
Role-Physical	0.76	0.02
Bodily Pain	0.66	0.10
General Health	0.53	0.27
Vitality	0.28	0.58
Social Functioning	0.34	0.39
Role-Emotional	0.07	0.59
Emotional Well-Being	-0.12	0.84
Correlation Between Factors	0.62	

n = 6,931

The factor scoring coefficients produced by the oblique factor solution produced fewer negative numbers than the factor scoring coefficients produced by the orthogonal factor solution used by Ware et al. [3]. For the physical health factor, only emotional well-being had a negative coefficient (-0.03); for the mental health factor, only physical functioning had a negative coefficient (-0.02). The magnitudes of the negative factor scoring coefficients are smaller than those derived in the orthogonal model (Table 3).

**Table 3:** Factor scoring coefficients* used to create SF-36 summary scores

	**Physical**		**Mental**	
	Orthogonal (PCS_uc_)	Oblique (PCS_c_)	Orthogonal (MCS_uc_)	Oblique (MCS_c_)
Physical Functioning	0.42	0.20	-0.23	-0.02
Role-Physical	0.35	0.31	-0.12	0.03
Bodily Pain	0.32	0.23	-0.10	0.04
General Health	0.25	0.20	-0.02	0.10
Vitality	0.03	0.13	0.24	0.29
Social Functioning	-0.01	0.11	0.27	0.14
Role-Emotional	-0.19	0.03	0.43	0.20
Emotional Well-Being	-0.22	-0.03	0.49	0.35

*Factor scoring coefficients are weights produced by the variables (the 8 scales) used to construct the factors (physical and mental). Factor scoring coefficients are based on factor loadings and vary by how much a particular variable contributes to the factor. Variables that correlate highly with a factor have large factor loadings and a larger factor score coefficient. Factor scoring coefficients are multiplied by the z-score for the scales and summed in order to obtain the estimated factor scores.

n = 6,931

### Sensitivity Analysis Results

As shown in Table 4, when the SF-36 physical health scale scores are 1 SD and the mental health scales are 0.3 SD above the mean, the PCS_uc _score is 62.2 (1.2 SD above the mean) and the MCS_uc _score is 49.6 (equal to the mean). As such, the MCS_uc _does not reflect the fact that the mental health scales are better than the mean. The alternative scoring algorithm results in a PCS_c _score that is 1 SD above the mean (60.0) and a MCS_c _score that is 0.5 SD above the mean (54.6). Similar results were found when the physical health scale scores were 0.3 SD above the mean and the mental health scale scores were 1 SD above the mean, resulting in a PCSuc score of 50.1 (at the mean) and a MCS_uc _score of 62.8 (1.2 SD above the mean). However, the alternate scoring algorithm produced a PCS_c _score of 55.1 and a MCS_c _score of 60.3 (0.5 SD and 1 SD above the mean, respectively).

**Table 4:** Hypothetical example comparing SF-36 orthogonal versus oblique scoring methods for the PCS and MCS

	Physical Health Scale z-scores = 1	Physical Health Scale z-scores = 0.3
	Mental Health Scale z-scores = 0.3	Mental Health Scale z-scores = 1
PCS_uc_	62.2	50.1
PCS_c_	60.0	55.1
MCS_uc_	49.6	62.8
MCS_c_	54.6	60.3

Summary score results if the scales that load heavily on physical health (physical health, role physical, bodily pain, general health) have z-scores of 1 and the scales that load heavily on mental health (vitality, social functioning, role-emotional and emotional well-being) have z-scores equal to 0.3. Also illustrated are the summary scores if the z-scores for scales loading heavily on physical health are equal to 0.3 and z-scores for scales loading heavily on mental health are equal to 1.

### Regression Analysis Results

Table 5 lists the SF-12 items, the variable names, the parameters estimated previously from the regression models where the orthogonal PCS_uc _and MCS_uc _were regressed on the SF-12 items and the parameters estimated here from regressing the obliquely derived PCS_c _and MCS_c _scores on the SF-12 items [2].

**Table 5:** Weights derived from orthogonal and oblique factor analysis used to create PCS-12 and MCS-12 scores

Item/Response Choice	**Variable**	**Physical Weight**		**Mental Weight**	
		Orthogonal	Oblique	Orthogonal	Oblique
**Moderate Activity (PF02)**		PCSuc-12	PCSc-12	MCSuc-12	MCSc-12
Limited a lot	PF02_1	-7.23216	-3.61039	3.93115+	0.21329+
Limited a little	PF02_2	-3.45555	-1.52769	1.86840+	0.15672+
**Climbing Several Flights of Stairs (PF04)**					
Limited a lot	PF04_1	-6.24397	-3.28556	2.68282+	0.12950+
Limited a little	PF04_2	-2.73557	-1.49769	1.43103+	0.08028+
**Accomplished less than you would like (RP2)**					
Yes	RP2_1	-4.61617	-3.72452	1.44060+	-0.67652
**Limited in the kind of work or activities (RP3)**					
Yes	RP3_1	-5.51747	-4.48695	1.66968+	-0.73255
**How much did pain interfere with norm work (BP2)**					
Extremely	BP2_1	-11.25544	-10.32862	1.48619+	-3.57055
Quite a bit	BP2_2	-8.38063	-7.60094	1.76691+	-2.24871
Moderately	BP2_3	-6.50522	-5.21603	1.49384+	-1.45064
A little bit	BP2_4	-3.80130	-2.76223	0.90384+	-0.85395
**In general, would you say your health is (GH1)**					
Poor	GH1_1	-8.37399	-6.90853	-1.71175	-4.28199
Fair	GH1_2	-5.56461	-4.56043	-0.16891	-2.78736
Good	GH1_3	-3.02396	-2.48820	0.03482+	-1.45741
Very Good	GH1_4	-1.31872	-1.09399	-0.06064*	-0.54378
**Have a lot of energy (VT2)**					
None of the time	VT2_1	-2.44706	-5.94178	-6.02409	-10.46333
A little of the time	VT2_2	-2.02168	-4.68268	-4.88962	-8.13254
Some of the time	VT2_3	-1.61850	-3.43746	-3.29805	-6.11303
A good bit of the time	VT2_4	-1.14387	-2.28701	-1.65178	-3.95386
Most of the time	VT2_5	-0.42251	-1.19645	-0.92057	-1.96823
**How much of the time health interferes w/social activities (SF2)**					
All the time	SF2_1	-0.33682*	-2.57689*	-6.29724*	-3.51605*
Most of the time	SF2_2	-0.94342*	-3.29868*	-8.26066*	-4.19005*
Some of the time	SF2_3	-0.18043	-2.42780	-5.63286	-3.20648
A little of the time	SF2_4	0.11038	-1.21560	-3.13896	-1.71673
**Accomplished less than you would like (RE2)**					
Yes	RE2_1	3.04365+	-0.27441	-6.82672	-3.37939
**Didn't do work or other activities as carefully as usual (RE3)**					
Yes	RE3_1	2.32091+	-0.87743	-5.69921	-3.38503
**Felt calm and peaceful (MH3)**					
None of the time	MH3_1	3.46638+	-0.64678	-10.19085	-9.27580
A little of the time	MH3_2	2.90426+	-0.47407*	-7.92717	-7.67490
Some of the time	MH3_3	2.37241+	-0.38979*	-6.31121	-5.60048
A good bit of the time	MH3_4	1.36689+	-0.53677*	-4.09842	-3.87498
Most of the time	MH3_5	0.66514+	-0.24474	-1.94949	-1.91559
**Felt downhearted and blue (MH4)**					
All of the time	MH4_1	4.61446+	-1.32335	-16.15395	-14.96225
Most of the time	MH4_2	3.41593+	-0.75981	-10.77911	-11.60997
A good bit of the time	MH4_3	2.34247+	-0.53385	-8.09914	-7.91401
Some of the time	MH4_4	1.28044+	-0.38595	-4.59055	-4.63416
A little of the time	MH4_5	0.41188+	-0.15932	-1.95934	-2.15359
**Constant**		56.57706	62.37966	60.75781	65.38813

* Magnitude Inconsistent/+ Incorrect Sign; n = 6,565

It is informative to compare the parameters estimated for the PCS_c_-12 and MCS_c_-12 to those estimated for the PCS_uc_-12 and MCS_uc_-12. Since the most favorable response choice for each item is the reference group, the y-intercept is the PCS-12 or MCS-12 score for a person who is in the best possible health (respondent selects the most positive response choice for all questions). Hence, the parameters estimated are decrements associated with each response choice for the items. For an individual item, response choices that represent a more favorable health state should have smaller decrements compared to a response choice for a less favorable health states such that we would expect negative coefficients in descending order of magnitude for the response choices of each item. The latter is not the case for four items in the PCS_uc_-12 model and five items in the MCS_uc_-12 model. In fact, the parameters estimated are positive, implying an increase in score, if the respondent chooses a non-favorable response choice over the most favorable response choices. These items are denoted with an asterisk ("*" or "^+^") in Table 5. In the PCS_c_-12 model, all parameters estimated were negative and in descending order of magnitude except for the response choices for two items (SF2 and EWB3). Similarly, in the MCS_c_-12 model, three items have higher estimates for less favorable response choices (PF02, PF04, and SF2). The magnitude of the weighting discrepancies are smaller than those obtained in the orthogonal model [2].

Correlations amongst the SF-36 and SF-12 summary measures are similar when the summary measure is derived using the correlated rather the uncorrelated algorithm. The correlation between PCSc and PCSc-12 was 0.98 whereas the correlation between the PCSuc and PCSuc-12 was 0.96. Similarly, the correlation between the MCSc and the MCSc-12 was slightly higher (0.97) than the correlation between the MCSuc and MCSuc-12 (0.96) (Table 6).

**Table 6:** Correlations among SF-36 and SF-12 summary measures

	PCS_uc_	PCS_c_	PCS_uc_-12	PCS_c_-12	MCS_uc_	MCS_c_	MCS_uc_-12	MCS_c_-12
PCS_uc_	1.00							
PCS_c_	0.91	1.00						
PCS_uc_-12	0.96	0.89	1.00					
PCS_c_-12	0.88	0.98	0.92	1.00				
MCS_uc_	-0.05	0.37	0.00	0.38	1.00			
MCS_c_	0.41	0.74	0.44	0.74	0.88	1.00		
MCS_uc_-12	0.01	0.40	0.02	0.41	0.96	0.86	1.00	
MCS_c_-12	0.42	0.74	0.45	0.76	0.85	0.97	0.89	1.00

n = 6,931

## Discussion

The SF-36 is one of the most commonly used HRQOL measures. Summary scores can be used to minimize problems with multiple comparisons. Ware et al. argue that the orthogonal method of developing summary scores is mathematically simpler and makes the interpretation of each scale less complicated compared to the oblique method [11,12]. However, several studies have shown that product-moment correlations between the physical and mental health factors range from 0.32 – 0.66, suggesting a moderate to strong correlation between the two components. [13] Summary scores that are forced to be uncorrelated may yield contradictory results compared to the scale scores. Our data demonstrate that this can be problematic if one assesses the significance of summary scores first and then assesses the scale scores only if the summary scores are significant. Alternatively, if the summary scores are presented alone, without the scale scores, the study may fail to detect an effect of an intervention or an important association with physical health, mental health or both. In fact, specific guidance regarding the SF-12 emphasizes the use of the summary scores because of the limitations of the 8 scale scores. [14,15] The present study suggests limitations of the summary scores need to be taken into account, as well.

This paper provides an alternative scoring algorithm for the SF-36 (version 1) and the SF-12 (version 1) physical and mental health summary scores. Our approach to constructing these scores is the same as the approach taken by Ware et al. [2,3] except we allow the physical and mental health constructs to be correlated. By allowing the constructs to be correlated, our results reduce the negative weights that were causing scale and summary score inconsistencies in the scoring algorithm for the uncorrelated SF-36 summary measures. Similarly, our approach reduced the positive weights in scoring algorithm for the uncorrelated SF-12 summary measures that result in weighting discrepancies. Thus, we conclude that by removing the constraint of "uncorrelated factors," it is likely the discrepancies between the scale and composite scores will be reduced.

While this manuscript focused on the method of composite score construction developed by Ware et al. [2,3], it is important to note that an alternative algorithm for the construction of correlated mental health and physical health summary measures exists [16,17]. The RAND-36 method is based on item response theory (IRT) scoring for scale scoring and uses only the 4 scales that are primarily indicative of physical health (physical functioning, role limitations due to physical health problems, pain, general health perceptions) and mental health (emotional well-being, role limitations due to emotional problems, social functioning, vitality), respectively, in creating the summary scores. Future research should also examine whether the RAND-36 method resolve inconsistent results between the SF-36 scale scores and the summary scores.

We recognize that there are several limitations inherent to this study. First, our sample includes only those receiving care from UMGA health plans, which may limit generalizability. When comparing the UMGA sample characteristics to those of the general population studied by Ware et al[2,3], there were some differences with respect to age, gender and race between the two samples [1,18]. Second, the majority of the study sites included in this study was from the West Coast which would also limit generalizability. Third, non-responders accounted for 41% of the patients contacted. As such, we do not know if the characteristics of the non-responders are the same as the responders. Hence, while this study derived weights based on one sample, we recommend that a similar approach be applied in other samples including the original sample from the general population that was used to generate the uncorrelated summary scores [18,19]. Lastly, even with the correlated factor solution, there are still some negative factor scoring coefficients.

## Conclusion

Summary scores that are forced to be uncorrelated may yield inconsistent results compared to the scale scores from which they are derived. This manuscript provides an alternative approach of deriving summary scores that allows the scores to be correlated. In this sample, the alternate scoring algorithm produced weights for scale scores and items that make it more likely that consistent results will be obtained for summary scores and scale scores. When presenting results from the SF-36 and SF-12 version 1, we recommend presenting the summary scores for the PCS_c _and MCS_c _derived from an obliquely rotated factor solution along with the scale scores and uncorrelated summary scores. Future research should be dedicated to deriving a scoring algorithm from an optimal correlated physical and mental health factor solution that is based on the general population, but the scoring algorithm presented in this manuscript can be employed until that is available. Lastly, we recommend that a similar approach be applied to derive summary measures for version 2 of the SF-36 and SF-12.

## Abbreviations

SF-12 Short-Form 12 Item Health Survey

SF-36 Short-Form 36 Item Health Survey

MCS_c _Mental Component Summary – Correlated

MCS_uc _Mental Component Summary – Uncorrelated

PCS_c _Physical Component Summary – Correlated

PCS_uc _Physical Component Summary – Uncorrelated

HRQOL Health-related Quality of Life

## Competing interests

The author(s) declare that they have no competing interests.

## Authors' contributions

Drs Cunningham, Farivar and Hays conceived of the study. Dr. Farivar wrote the first draft. All authors wrote and contributed sections of the manuscript. Dr. Farivar performed the statistical analysis. All authors read and approved the final version of the manuscript.
